# The Clinical Implications of Anti-thyroid Peroxidase Antibodies in Graves’ Disease in Basrah

**DOI:** 10.7759/cureus.36778

**Published:** 2023-03-28

**Authors:** Emad S Alhubaish, Nassar T Alibrahim, Abbas A Mansour

**Affiliations:** 1 Diabetes and Endocrinology, Faiha Specialized Diabetes, Endocrine and Metabolism Center, University of Basrah, Basrah, IRQ

**Keywords:** antithyroid drug, graves relapse, giraffe appearance, thyroid peroxidase antibodies, graves’ disease

## Abstract

Background

Graves’ disease (GD) is an autoimmune disease, with thyrotropin receptor antibodies (TRAbs) being the most important cause in the pathogenesis. The aim of this study is to assess the clinical significance of anti-TPO Abs in GD.

Methods

A retrospective study was conducted at the Faiha specialized Diabetes, Endocrine, and Metabolism Center (FDEMC) in Basrah during the period between December 2021 and December 2022. A total of 141 patients with GD were involved in this study, and of them, 97 (68.8%) were women. They were divided into two groups: patients with positive and negative anti-TPO Abs groups.

Results

Positive anti-TPO Abs were seen in 83 patients (58.9%) with GD. Pretreatment-free thyroxine level (ng/dL) was higher in the anti-TPO Abs positive GD patients than in those with negative antibodies (3.7±0.2 versus 3.0±0.2 with a p=0.021). Similarly, higher TRAb titers (IU/ml) at baseline were also seen in patients with positive anti-TPO Abs (9.8±0.7 versus 6.8±0.8) with a p=0.008. Giraffe appearance on thyroid ultrasound was more common in the group with positive anti-TPO Abs as compared to patients with negative anti-TPO Abs: 20 (87.0%) versus 3 (13.0%) with a p=0.005. A higher anti-TPO Abs titer (IU/mL) was associated with a baseline TRAb level of more than 6.4 IU/mL, and giraffe appearance on thyroid ultrasound (206.5±20.0 p-value<0.0001 and 228.0±35.3 p value=0.007, respectively).

Conclusion

A positive anti-TPO Abs in GD is associated with a high TRAb titer and free T4 level at baseline, as well as a giraffe appearance on thyroid ultrasound.

## Introduction

Graves’ disease (GD) is the most common cause of hyperthyroidism, with an incidence of 20 to 50 cases per 100,000 persons per year [1]. The most prevalent age for the presentation of the disease is between 30 and 50 years; however, all age groups may be affected. It has been found that about 3% of women and 0.5% of men are affected by this disease during their lifespan [2]. Antibodies of the IgG1 type directed against the thyroid-stimulating hormone (TSH) receptors are both specific for and crucial in the pathogenesis of GD [3]. These antibodies, produced mainly by intrathyroidal B cells, reflect the disease’s primary autoimmune reaction as well as stimulate uncontrolled thyroid hormone production by the hypothalamic-pituitary-thyroid axis [4]. In addition, antibodies directed against thyroglobulin (TG) and thyroid peroxidase (TPO) are also frequently present in GD, and antigen spreading leads to the appearance of such antibodies that are not known to be pathogenic based on prior studies [5].

Thyroid-stimulating hormone receptor antibodies (TRAbs), which are prevalent in autoimmune thyroid disease (AITD), are found in 90% (and even higher when using newer generations of assay methods) of patients with Graves’ disease, 10-75% of patients with atrophic thyroiditis, and 20% of patients with Hashimoto thyroiditis (HT) [6]. Moreover, relapse becomes highly likely within two years if TRAbs are present at the end of GD treatment [7].

TPO is a membrane-bound enzyme responsible for the iodination of tyrosyl residues in the thyroglobulin molecule [8]. It is known as the microsomal antigen due to its intracellular location. TPO activity is not blocked by anti-TPO Abs in healthy individuals, nor do they interfere with the blocking activity of anti-TPO Abs in patients with AITD [9]. While anti-TPO Abs in patients with AITD can fix complement, destroy thyroid cells, and competitively inhibit enzymatic activity [10], the risk factors for anti-TPO Abs presence in GD include a higher age, iodine deficiency or excess, a family or personal history of AITD, along with idiopathic and autoimmune causes. Furthermore, paradoxically, smoking and pregnancy appear to decrease the risk of positive anti-TPO Abs [11].

Anti-TPO Abs are more prevalent and more indicative of thyroid disease than anti-thyroglobulin antibodies (anti-TG Abs) [12]. In AITD patients, anti-TPO Abs are found in about 90-95% of patients, while being present in about 80% of GD patients and only in 10-15% of non-AITD patients [6]. Anti-TPO Abs in Hashimoto's thyroiditis play a role in thyroid cell damage; however, they do not play an established role in GD [13]. Anti-TG Abs do not cause thyroid cell destruction and can be detected in about 10% of healthy young subjects and 15% of patients who are greater than 60 years of age, as well as in about 60-80% of patients with Hashimoto's thyroiditis and 50-60% of patients with GD [6].

In GD patients treated with antithyroid drugs (ATD) that depict immunomodulatory effects, there is evidence supporting the decrease in TRAbs and anti-TPO Abs titers [14]. Long-term ATD treatment as compared to the conventional 18-24-month treatment regimen for GD, female gender, nonsmoking, and absence of thyroid eye disease (TED) was associated with a higher remission rate [15,16]. Positive anti-TPO Abs, when present in GD, are associated with a modest reduction in the relapse rate following ATD treatment. The odds for sustained remission significantly increase with positive anti-TPO Abs following the withdrawal of ATDs in newly diagnosed GD [14].

With moderate TRAbs elevation in GD (6-10 IU /mL), the addition of anti-TPO Abs measurement six months after diagnosis could predict GD relapse [17]. Anti-TPO Abs clinical significance in TED is not yet clarified, as some studies in children demonstrated an association of TED with certain anti-TPO Abs polymorphisms, while other studies in adults revealed that TED was associated with a low level of anti-TPO Abs titer [18]. Anti-TPO-Abs presence in GD could indicate a more aggressive disease that requires longer treatment, especially in the young female population [19]. Studies present that low titers of anti-TPO Abs in patients with GD and high TRAbs levels at the time of diagnosis are associated with TED [20]. Therefore, the aim of this study was to assess the clinical significance of anti-TPO Abs in GD.

## Materials and methods

Design and settings

A retrospective study was conducted at the Faiha specialized Diabetes, Endocrine, and Metabolism Centre (FDEMC) in Basrah from December 2021 to December 2022. A total of 141 patients with GD were included in the study, of whom 97 (68.8%) were women. The patients were divided into two groups: positive and negative anti-TPO-Abs. Informed verbal consent was obtained from each participant after explaining the aim of the study in accordance with the ethical standards of the FDEMC Research Committee, from which the ethical approval was obtained under reference no. 21/43/67, and with the 1964 Declaration of Helsinki and its later amendments or comparable ethical standards.

Inclusion and exclusion criteria

All patients with GD and TRAbs positive were included in this study. TRAbs-positive patients without GD, TRAbs-negative patients with GD, patients with incomplete data, and patients with TED and normal thyroid function or hypothyroidism were excluded from this study.

Data collection

A detailed history was taken from all, including age, gender, and family history, which was considered positive when at least one first-degree relative suffered from goiter, hypothyroidism, or hyperthyroidism. We examined the patient’s anthropometrics, including weight, height, and body mass index (BMI). Thyroid eye disease was also assessed according to Graves's orbitopathy severity assessment and the clinical activity score element by subdividing patients into those with and without TED.

Biochemical assays

Thyroid function tests and thyroid autoantibodies were measured by the Roche® electrochemiluminescence method. Anti-TPO-Abs titer of more than 34 (IU/mL) was considered positive. Normal reference values were as follows: TSH: 0.27-4.2 μIU/mL, free thyroxine (free T4): 0.93-1.7 ng/dL, TRAbs: ≤1.75 IU/mL, and anti-TG Abs: <115 IU/mL [21].

Patients with GD who encountered a hypothyroid state while on the usual ATD regimen were recognized and studied. According to the duration of ATD treatment, patients were divided into two groups: those treated for one to two years and those treated for more than two years (long-term treatment) [22].

Regarding the final state of the patients following the diagnosis and treatment, patients were divided into those who underwent surgery or continued on ATD treatment and those who had relapsed or gone into remission after surgery or ATD. Relapse is defined as the reappearance of symptoms and biochemical thyroid dysfunction. Remission was considered to be the time when neither symptoms nor an impaired thyroid function test were present following the withdrawal of ATD for more than six months [23].

We performed thyroid ultrasonography for each patient and classified the findings into four groups according to the sonographic pictures, which were then reviewed by two expert sonographers to observe diffuse goiter (homogeneous echogenicity), nodular goiter (having definite nodule(s) in one or both thyroid lobes), giraffe appearance, and heterogeneous goiter with pseudo-nodularity (inhomogeneous echogenicity with small nodules-like appearance without definite wall).

All patients were sent for a dual-energy X-ray absorptiometry scan, and the patients were classified into two groups: normal bone mineral density (T score ≥-1) and reduced bone mineral density, which was further included in osteopenia (T score -1 to -2.5) and osteoporosis (T score ≤−2.5). The T-score was used for patients who were aged 50 and above, while the Z-score was used for patients below 50 years of age [24].

Statistical analysis

All data were tabulated using the Statistical Package for Social Sciences (SPSS) version 25 (IBM SPSS, Armonk, NY). A comparative analysis was done between different groups. The chi-square test was used for categorical variables, while the t-test and Mann-Whitney test were used for continuous variables when appropriate. Results with a p-value <0.05 were considered to have reached the level of statistical significance. Receiver operator characteristics (ROC) statistics were used to calculate various cut-offs, the area under the curve, odds, sensitivity, and specificity.

## Results

**Figure 1:**
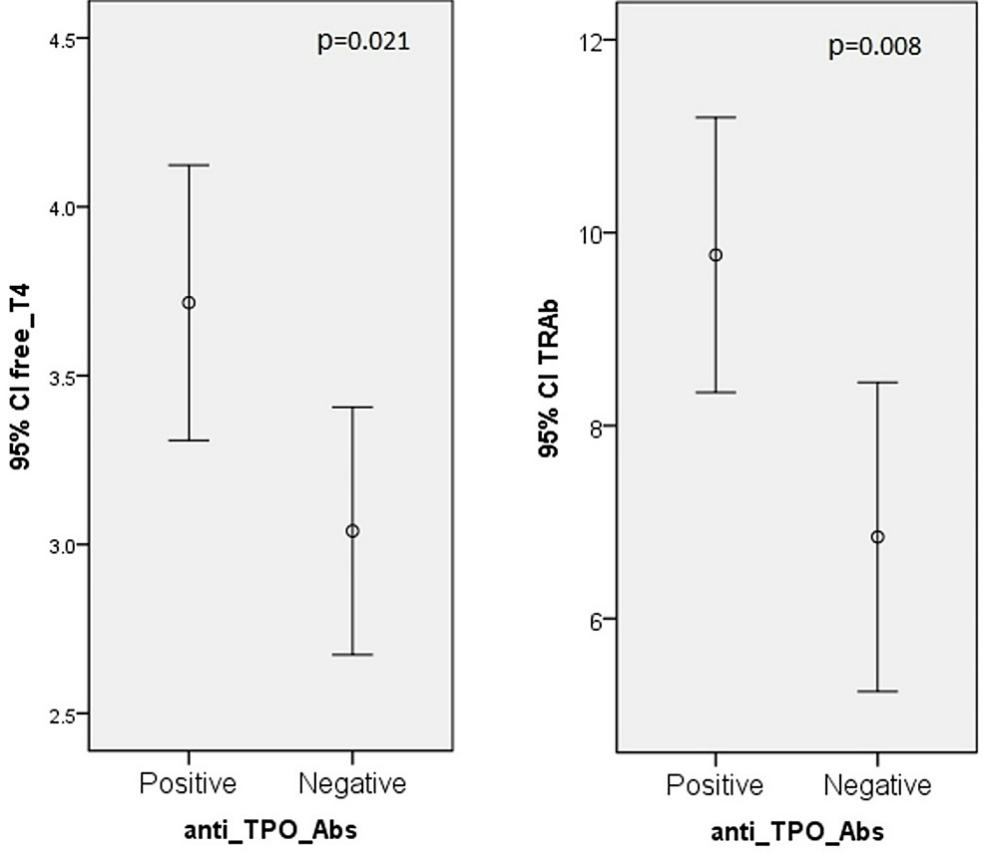
The 95% confidence intervals of free T4 (Left) and TRAb (right) in patients with both positive and negative anti-TPO Abs.

The basic characteristics of the 141 patients with GD in this study are shown in Table 1. The mean age was found to be 42.3±1.2 years, and 97 (68.8%) participants were female. The mean anti-TPO-Abs titer was 154.5±13.4 IU /mL, and 83 (58.9%) patients had positive anti-TPO Abs. The predictors of positive anti-TPO-Abs can be assessed in Table 2. Patients with positive anti-TPO-Abs demonstrated a higher baseline free T4 (ng/dL) level than those with negative antibodies (3.7±0.2 versus 3.0±0.2, p=0.021). A higher level of TRAbs (IU/mL) titer at baseline was also seen in patients with positive anti-TPO Abs (9.8±0.7 versus 6.8±0.8, p=0.008), as seen in Figure 1.

**Table 1:** General characteristics of the patients (N=141) ATD: antithyroid drug, anti-TG Ab: antithyroglobulin antibody, anti-TPO Abs: antithyroid peroxidase antibody, TRAb: thyrotropin receptor antibody, free T4: free thyroxine, BMD: bone mineral density, TED: thyroid eye disease, BMI: body mass index, TSH: thyroid-stimulating hormone.

Variables	Categories	Mean±SD or N (%)
Age (years)		42.3±1.2
Gender	Men	44(31.2)
	Women	97(68.8)
Duration of the disease (years)	≤5	80(56.7)
	>5	61(43.3)
Family history of thyroid diseases	Yes	66(46.8)
	No	75(53.2)
BMI	Obese	54(38.3)
	Non-obese	87(61.7)
TED	Yes	54(38.3)
	No	87(61.7
TSH (µIU/mL)	Mean	0.007±0.0008
	>0.005	17(12.1)
	≤0.005	124(87.9)
Baseline free T4 (ng/dL)	Mean	3.4±0.1
Anti-TPO Abs (>34 IU/mL)	Mean	154.5±13.4
	Positive	83(58.9)
	Negative	58(41.1)
TRAbs (IU/ML)	Mean	8.6±0.5
Anti-TG Ab (IU/mL)	Positive	58(41.1)
	Negative	83(58.9)
BMD	Normal	95(68.4)
	Reduced	46(32.6)
Thyroid ultrasound	Diffuse	30(21.3)
	Nodular	19(13.5)
	Giraffe	23(16.3)
	Pseudo nodularity	69(48.9)
Treatment	ATD only	129(91.5)
	ATD + surgery	12(8.5)
ATD duration (years)	1-2	67(47.5)
	>2	74(52.5)
Hypothyroidism during treatment	Yes	45(31.9)
	No	96(68.1)
Fate	On ATD	80(56.7)
	Remission	12(8.5)
	Relapse	40(28.4)
	Hypothyroidism	9(6.4)

**Table 2:** Characteristics of patients with positive versus negative anti-TPO Abs. TSH: thyroid-stimulating hormone, Anti-TG Ab: antithyroglobulin antibody, anti-TPO Abs: antithyroid peroxidase antibody, TRAbs: thyrotropin receptor antibody, free T4: free thyroxine. Data are expressed either as mean±SD or N (%).

		Anti-TPO Abs positive patients	Anti-TPO Abs negative patients	P-value
Mean age (in years)		40.5±1.5	44.5±1.7	0.095
	≥45 years	39(47.0)	31(53.4)	0.450
	<45 years	44(53.0)	27(46.6)	
Gender	Men	24(28.9)	20(34.5)	0.483
	Women	59(71.1)	38(65.5)	
Family history of thyroid disease	Yes	37(44.6)	29(50.0)	0.526
	No	46(55.4)	29(50.0)	
Body mass index	Obese	31(37.3)	23(39.7)	0.782
	Non-obese	52(62.7)	35(60.3)	
TSH (µIU/mL)		0.008±0.001	0.007±0.001	0.724
Free T4 (ng/dL)		3.7±0.2	3.0±0.2	0.021
TRAbs (IU/mL)		9.8±0.7	6.8±0.8	0.008
Anti-TG Ab (IU/mL)	Positive	37(44.6)	21(36.2)	0.320
	Negative	46(55.4)	37(63.8)	
Thyroid ultrasound	Diffuse	12(14.5)	18(31.0)	0.005
	Nodular	9(10.8)	10(17.2)	
	Giraffe	20(24.1)	3(5.2)	
	Pseudo nodularity	42(50.6)	27(46.6)	

**Figure 2:**
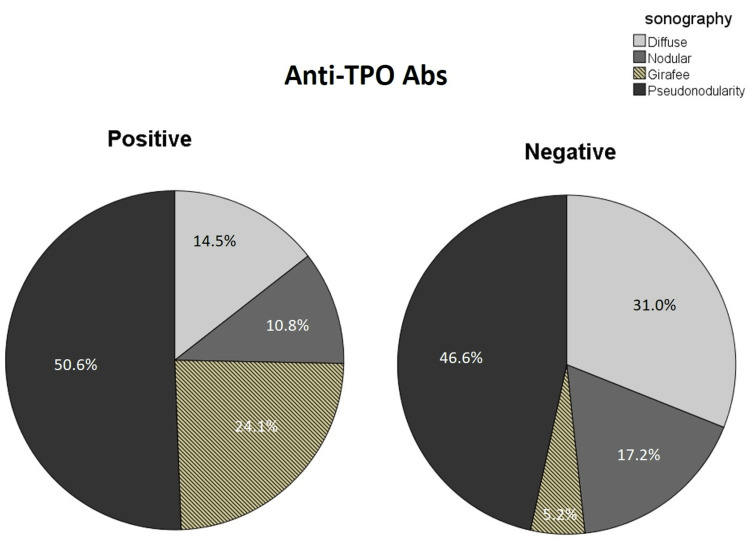
Distribution of ultrasonographic features according to anti-TPO Abs state.

Twenty patients (87%) with giraffe appearance on thyroid ultrasonography demonstrated positive anti-TPO-Abs, while only three patients (13%) showed negative anti-TPO Abs (p=0.005). Gender, age, BMI, and family history of thyroid disease could not be assessed to predict the positivity of anti-TPO Abs in patients with Graves’ disease. Pseudonodularity appeared to be the most common ultrasonographic feature in both positive and negative anti-TPO Abs patients, at 42 (60.6%) and 27 (46.6%), respectively. However, the percentage of giraffe appearance increased significantly in patients with positive anti-TPO Abs: 20 (24.1%) versus 3 (5.2%) in anti-TPO Abs negative patients (p=0.005), as seen in Figure 2.

In Table 3, the factors impacting the anti-TPO-Abs titer can be found. Baseline TRAbs titers higher than 6.4 IU/mL were significantly associated with higher anti-TPO Abs titers (p<0.0001). Moreover, giraffe ultrasound thyroid features were found to be associated with a higher titer of anti-TPO Abs (p=0.007) compared to other ultrasound thyroid features.

**Table 3:** Factors affecting anti-TPO Abs titer. TSH: thyroid-stimulating hormone, anti-TG Ab: antithyroglobulin antibody, anti-TPO Ab: antithyroid peroxidase antibody, TRAbs: thyrotropin receptor antibody, free T4: free thyroxine.

	Categories	Anti-TPO Abs titer mean±SD	P-value
Age (years)	≥45	151.0±20.0	0.799
	<45	157.8±17.9	
Gender	Men	145.4±24.2	0.649
	Women	157.6±16.1	
Family history of thyroid disease	Yes	147.7±19.1	0.635
	No	160.4±18.7	
Body mass index	Obese	142.1±19.0	0.467
	Non-obese	162.1±18.2	
TSH (µIU/mL)	>0.005	169.3±34.3	0.683
	≤0.005	152.4±14.5	
Free T4 (ng/dL)	≥2.7	171.9±19.8	0.178
	<2.7	135.8±17.6	
TRAbs (IU/mL)	>6.4	206.5±20.0	<0.001
	≤6.4	103.1±15.6	
Anti-TG Ab (IU/mL)	Positive	158.3±21.0	0.810
	Negative	151.7±17.4	
Thyroid ultrasound	Diffuse	95.0±19.3	0.007
	Nodular	102.8±28.6	
	Giraffe	228.0±35.3	
	Pseudo nodularity	170.0±20.6	

While positive anti-TPO Abs did not significantly impact or predict GD outcomes such as disease duration, TED, bone mineral density, ATD treatment duration, the development of hypothyroidism during therapy, or the rate of relapse or remission in GD, as seen in Table 4, we further proceeded with our data analysis using the ORC statistics to search for cut-off values of anti-TPO Abs titers (IU/mL) that may depict some level of association with GD outcomes (Table 5).

**Table 4:** Effect of anti-TPO Abs positivity with outcomes of GD. Anti-TPO Abs: antithyroid peroxidase antibody, GD: Grave’s disease, TED: thyroid eye disease, BMD: bone mineral density, ATD: antithyroid drug.

Variables	Categories	Anti-TPO positive Abs	Anti-TPO negative Abs	P-value
Duration of disease (years)	≤5	52(62.7)	28(48.3)	0.090
	>5	31(37.3)	30(51.7)	
TED	Yes	28(33.7)	26(44.8)	0.182
	No	55(66.3)	32(55.2)	
BMD	Normal	54(65.1)	41(70.7)	0.483
	Reduced	29(34.9)	17(29.3)	
Treatment	ATD only	78(94.0)	51(87.9)	0.206
	ATD + surgery	5(6.0)	7(12.1)	
ATD duration (years)	1-2	39(47.0)	28(48.3)	0.880
	>2	44(53.0)	30(51.7)	
Hypothyroidism while on treatment	Yes	27(32.5)	18(31.0)	0.851
	No	56(67.5)	40(69.0	
Fate	On ATD	50(60.2)	30(51.7)	0.352
	Remission	6(7.2)	6(10.3)	
	Relapse	24(28.9)	16(27.6)	
	Hypothyroidism	3(3.6)	6(10.3)	

**Table 5:** Anti-TPO-Abs titer cut-off associated with some diseases’ outcomes. Anti-TPO Abs: antithyroid peroxidase antibody, GD: Grave’s disease, OR: odds ratio, TED: Thyroid eye disease, ATD: antithyroid drugs.

		Cut-off (IU/mL)	OR	Sensitivity%	Specificity%	P-value
Duration of disease >5 years		≤5.4	3.735	16	95	0.025
Thyroid eye disease		≤25.9	2.031	41	75	0.054
Treatment	Surgery	≤40	2.868	67	59	0.087
Hypothyroidism while on treatment		≤5	3.353	16	94	0.040
Fate	Still on ATD	≥287	2.571	25	89	0.043
	Hypothyroidism after surgery	≤20	6.8	67	77	0.003
	Remission	≤16	3.474	42	83	0.038
	Relapse: When anti-TPO Abs titer					
	<35 IU/mL	≥26	5	50	83	0.010
	≥35 IU/mL	≤170	4.2	54	78	0.004

The longer the duration following the diagnosis, the lower the titer of anti-TPO Abs (titer ≤5.4 IU/mL; odd ratio = 3.735, sensitivity = 16%, specificity = 95%, p=0.025). Those patients who developed hypothyroidism while on treatment were observed to have low anti-TPO Abs titers (≤5 IU/mL; odd ratio = 3.353, sensitivity = 16%, specificity = 94%, p=0.040). Likewise, hypothyroidism following surgery for Graves’ disease was more likely to occur with an anti-TPO Abs titer of ≤20 IU/mL (odd ratio = 6.8, sensitivity = 67%, specificity = 77%, p=0.003). Patients in remission also presented low anti-TPO Abs titers (≤16 IU/mL; odd ratio = 3.474, sensitivity = 42%, specificity = 83%, p=0.038).

Anti-TPO Abs titers greater than 26 IU/mL (odd ratio = 5, sensitivity = 50% specificity = 83%, p=0.010) and less than 170 IU/mL (odd ratio = 4.2, sensitivity = 54% specificity = 78%, p=0.004) tend to be associated with a higher relapse rate. TED and definitive treatment with ATD or surgery do not appear to be associated with an anti-TPO Abs titer level.

## Discussion

Graves’ disease is a common AITD with variable clinical courses as well as the production of a high number of antibodies, of which anti-TPO Abs depict no established clinical significance [5]. The higher percentage of women in comparison to men involved in this study reflects the well-known nature of the disease [2]. The fact that more than half of the patients continue to be on ATD for more than 24 months can be attributed either to the persistence of the disease or the relapse, which was seen in more than a quarter of the patients. The timing of thyroid surgery for patients who underwent it was variable throughout the course of the disease; some were done at the early stages of the disease, while others were conducted after two or more years of diagnosis.

In this study, we aimed to focus on GD outcomes associated with positive anti-TPO Abs that could aid in the management of GD. In a study conducted by Carvalho et al., anti-TPO Abs were present in about 80% of patients with Graves’ disease [6]. However, the anti-TPO Abs in this study were positive in only about 58.9% of the samples, which may be due to the small sample size. Likewise, we found that a positive anti-TPO Abs state could not be predicted by age or gender, despite previous studies suggesting that older age and a positive family history were risk factors for anti-TPO Ab positivity [11].

In a retrospective study, Anagnostis et al. identified that remission in patients with GD treated with ATDs can only be independently predicted through the duration of the first ATDs course of more than 24 months. Female gender, non-smoking, the absence of TED, iatrogenic hypothyroidism, and lower free-T4 after six months of therapy were associated with a longer duration of remission [16]. Azizi et al. found an increased remission rate with longer-term ATD treatment [15]. They demonstrated positive anti-TPO Abs to be associated with high baseline free T4, which could indirectly be against remission. Additionally, in their study, the positive anti-TPO Abs were not found to be associated with TED or the duration of ATD treatment. In this study, we found that low titers of anti-TPO Abs were associated with hypothyroidism during treatment and after surgery, as well as with the remission rate.

Struja et al., in a metanalysis, studied factors that could predict relapse following the withdrawal of ATDs. They found that TED, smoking, thyroid volume measured by sonography (goiter size), free-T4 level, and TRAbs titer were significantly associated with relapse, whereas male gender and age did not show significant associations [25]. In this study, positive anti-TPO Abs were associated with high TRAbs titers in addition to the association found with giraffe appearance on thyroid ultrasound.

To the best of our knowledge, this is the first study to correlate the morphological ultrasound features with the anti-TPO Abs state and titer in GD. Although the pseudo-nodularity feature was the most prevalent in all GD patients, when anti-TPO Abs positivity is considered, the giraffe appearance becomes a discriminating factor in patients with anti-TPO Abs negativity. A future study powered toward this aspect may help in addressing this phenomenon.

One study demonstrated positive anti-TPO Abs associated with aggressive GD and longer time to treat, especially in young female patients [19]. Moreover, no correlation was found between anti-TPO Abs positivity and GD outcomes, such as disease duration. The remission rate was associated with low titers of anti-TPO Abs, whereas in our study, the relapse rate was associated with variable anti-TPO Abs titers.

A few studies showed that anti-TPO Abs titers decreased with ATD treatment and returned to their basal level after ATD withdrawal [26,27]. Other studies revealed that with an increase in anti-TPO Abs titer during ATD therapy in anti-TPO Abs positive patients at the time of diagnosis, the relapse is very likely [23]. However, we found that anti-TPO Abs were not correlated with ATD treatment, but longer-term ATD treatment of more than five years was associated with low titers of anti-TPO Abs.

It was further observed that hypothyroidism prevails in patients with Graves’ disease undergoing partial thyroidectomy in the setting of positive anti-TPO Abs [28]. In this study, a significant association was observed between patients who were hypothyroid while on ATD and those who were hypothyroid after Graves’ disease surgery with low titers of anti-TPO Abs.

In a meta-analysis of over 54 trials [25], pretreatment-free T4 and TRAbs levels were significantly associated with relapse risk. In this study, we did not find an association between anti-TPO Abs positive patients and relapse outcome in Graves’ disease, despite the fact that pretreatment-free T4 and TRAbs levels significantly predicted positive anti-TPO Abs in patients with Graves’ disease.

Relapse rates have been shown to decrease when prolonged ATD treatment is used for more than 24 months [16]. With a recently published randomized controlled trial and a comparison with the standard 18 to 24 months course, a more prolonged ATD treatment of around 95 months was found to be superior to the standard treatment, achieving only a 15% relapse rate [15].

Thyroid peroxidase can be expressed in the orbital tissues of TED patients. Data are conflicting regarding the association of anti-TPO Abs and TED. Some conducted studies in pediatric patients show a positive correlation between high anti-TPO Abs levels and TED [29]. Studies in adults are more obscure, as some have implied a protective role of anti-TPO Abs in TED as there is an association between low anti-TPO Abs titers and TED. However, the majority of studies did not find any difference in anti-TPO Abs levels between patients with and without TED [20].

It was found that anti-TPO Abs levels were correlated with IgG4 levels, which increase in TED patients compared to patients without TED. In contrast, anti-TPO Abs levels demonstrated no difference between TED patients with high and normal IgG4 levels [26]. The role of anti-TPO Abs in TED remains unclear due to the low number of studies and their controversial results, therefore requiring further investigation. In this study, no association between TED and anti-TPO Abs-positive GD was observed. Bone mineral density, along with a family history of thyroid disease, was not associated with positive anti-TPO Abs, and no studies could be identified to compare this phenomenon.

The main limitation of this study is the retrospective design. Anti-TPO Abs was done only at the time of diagnosis, and several patients were excluded due to lost follow-up. In addition, the relatively small number of participants in the study was also another limitation. We did not consider other clinical factors such as smoking status and goiter size. TRAbs levels at the cessation of ATD treatment were also not assessed for all patients.

## Conclusions

In patients with GD, more than half of the patients had a positive anti-TPO Abs. A correlation was found between positive anti-TPO Abs and high TRAbs titers, the free T4 level at baseline, and the giraffe appearance on thyroid ultrasound. Further studies should examine this association to achieve more accurate results. Although the state of anti-TPO Abs being positive or negative was associated with GD outcomes, defining new titers for each outcome, such as relapse and the development of hypothyroidism, may aid in future predictions.
